# Expression characteristic of CXCR1 in different breast tissues and the relevance between its expression and efficacy of neo-adjuvant chemotherapy in breast cancer

**DOI:** 10.18632/oncotarget.16893

**Published:** 2017-04-06

**Authors:** Miao-Qun Xue, Jun Liu, Jian-Feng Sang, Lei Su, Yong-Zhong Yao

**Affiliations:** ^1^ Department of General Surgery, Nanjing Drum Tower Hospital The Affiliated Hospital of Nanjing University Medical School, Nanjing 210008, China; ^2^ Department of General Surgery, The Jiang Bei People's Hospital of Nanjing, Nanjing 210048, China

**Keywords:** breast cancer, breast cancer stem cells, chemokine receptor, CXCR1, neo-adjuvant chemotherapy

## Abstract

**Objective:**

To investigate chemokine receptor CXCR1 expression characteristic in different breast tissues and analyze the relationship between CXCR1 expression changes in breast cancer tissue and efficacy of neo-adjuvant chemotherapy.

**Results:**

Chemokine receptor CXCR1 was lowly expressed in normal breast tissues and breast fibroadenoma, but highly expressed in breast cancer. It was significantly correlated with pathological stage, tumor cell differentiation, and lymph node metastasis (*P* < 0.05). After neo-adjuvant chemotherapy, CXCR1 expression in breast cancer tissues decreased. Among these 104 breast cancer patients with different molecular subtypes, the survival rate with Luminal A was the highest, followed by the Luminal B breast cancer, TNBC was the worst.

**Materials and Methods:**

104 cases with breast carcinoma, 20 cases with normal breast and 20 cases with breast fibroadenoma were included and followed up. Immunohistochemistry was used to detect the expression of CXCR1 in the various tissues. The relationship between the CXCR1 expression changes in breast cancer biopsies and surgical specimens, as well as the efficacy of neo-adjuvant chemotherapy, was analyzed.

**Conclusions:**

Chemokine receptor CXCR1 could be used as an indicator to predict benign or malignant breast disease, and it can even predict the malignancy degree of breast cancer, as well as its invasive ability and prognosis.

## INTRODUCTION

Breast cancer is one of the common malignant tumors in women, which has received increasing attention [1]. Neo-adjuvant chemotherapy was a new progress in the comprehensive therapy of breast cancer. Pathological characteristic changes in primary tumors before and after chemotherapy could accurately determine the efficacy of neo-adjuvant chemotherapy, which is advantageous for clinical research. Current studies had found many chemokines and receptors relating to the occurrence and development of breast cancer such as CXCR4, CCR5 and CCL12. Recently the chemokine receptor CXCR1 has also been reported to have an important role in the progression of breast cancer [2–4].

In this study, we observed the expression of CXCR1 in normal breast tissues, breast fibroadenoma tissues and breast cancer tissues to see its expression in different breast diseases and detected the expression changes of CXCR1 in breast cancer tissues before and after neo-adjuvant chemotherapy in patients with breast cancer, in order to explore its correlation with the efficacy of neo-adjuvant chemotherapy.

## RESULTS

### Expression of CXCR1 in different breast tissues

**Figure 1:**
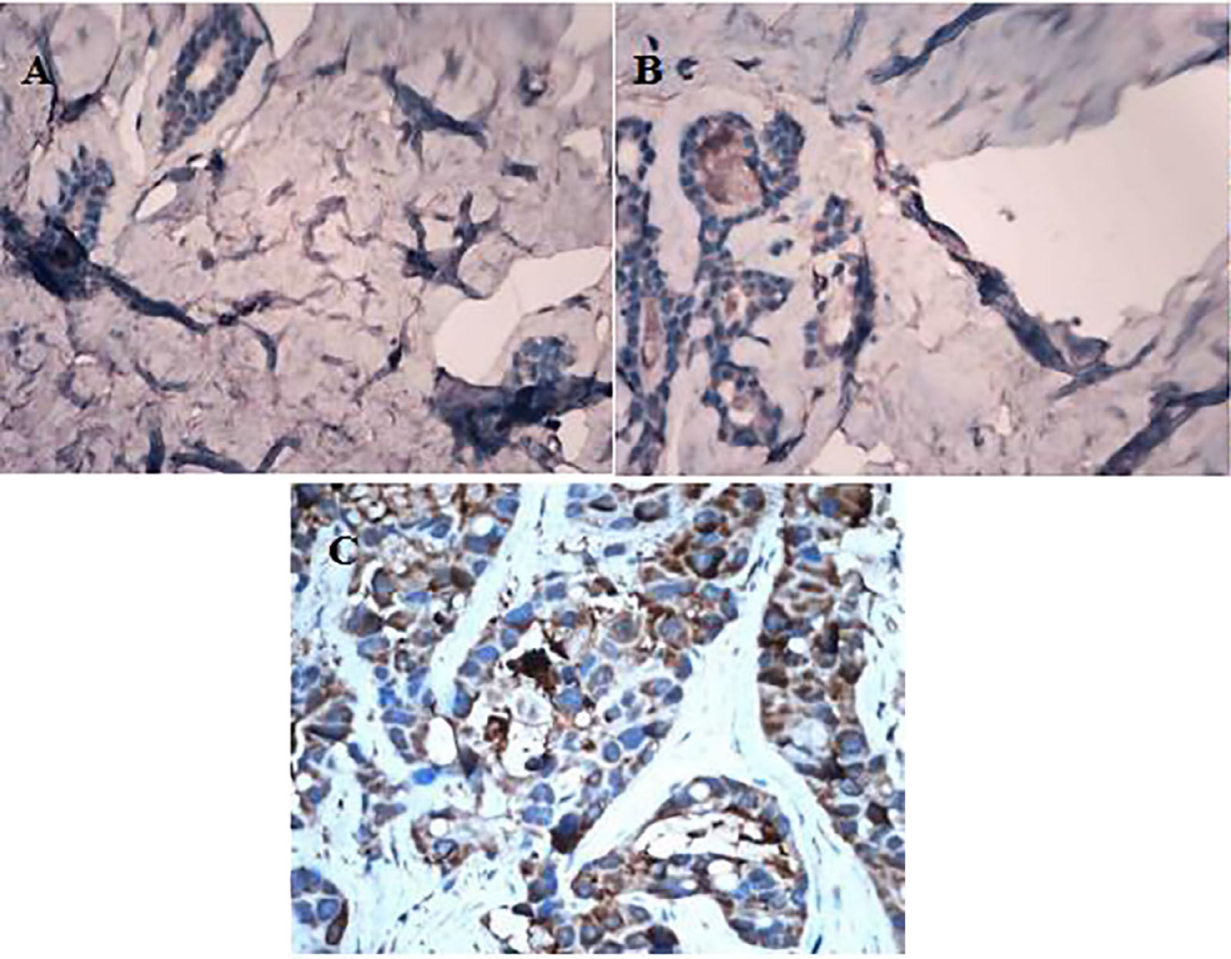
Different expression of CXCR1 in normal breast tissue, breast fibroadenoma and breast carcinoma tissue (IHC, ×400) (**A**) CXCR1 expression in normal breast tissues; (**B**) CXCR1 expression in breast fibroadenoma tissue; (**C**) CXCR1 expression in breast carcinoma tissue.

**Figure 2:**
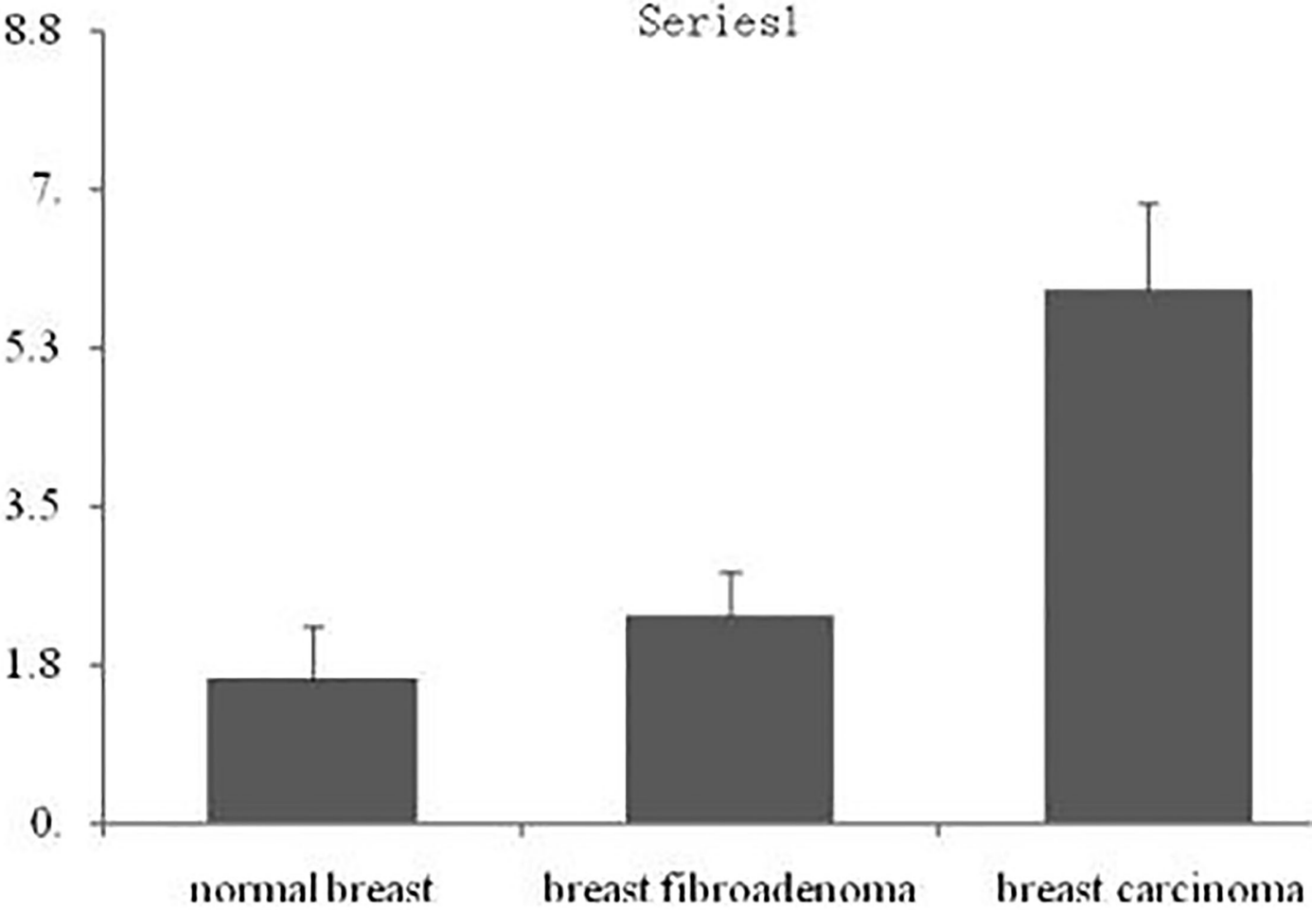
Integral analysis of CXCR1 expression in breast cancer tissues Note: *P < 0.01, compared with normal breast tissues; ^#^*P* < 0.01, compared with breast fibroadenoma tissues.

Chemokine receptor CXCR1 was expressed in different degrees in normal breast tissues, breast fibroadenoma and breast cancer tissues (Figure 1). It was lowly expressed in normal breast tissue and breast fibroadenoma, but highly expressed in breast cancer tissues. In addition, CXCR1 was expressed in almost all breast cancer cells, mainly expressed in the cytoplasm of tumor cells, but rarely expressed in the nucleus. The PI of CXCR1 in normal breast tissue, breast fibroadenoma and breast carcinoma were 1.6 ± 0.57, 2.3 ± 0.48 and 5.9 ± 0.95 respectively; which gradually increased. Differences among PIs in normal breast tissues and breast fibroadenoma were not statistically significant (*P* > 0.05), while the difference among PIs in breast cancer tissues were significantly higher than that in the other groups (*P* < 0.01) (Figure 2).

### The relationship between the expression of CXCR1 in breast carcinoma tissues and various clinical parameters of patients

The expression of CXCR1 in breast tumor tissues revealed no correlation with age and primary tumor size (*P* > 0.05), but revealed a significant difference with pathological stage, differentiation degree, lymph node metastasis, the status of the hormone receptor, and Her2 expression (*P* < 0.05). For the different pathological stages, there was no difference in the expression of CXCR1 between stage I and stage II (*P* > 0.05), but statistically different between stage II and stage III, also statistically different between stage I and stage III (*P* < 0.05). In terms of differentiation degree among sub-groups, no difference was found in the expression of CXCR1 between the highly differentiated group and moderately differentiated group (*P* > 0.05). On the contrary, statistical difference was found between the highly differentiated group and mildly differentiated group, as well as between the mildly differentiated group and moderately differentiated group (*P* < 0.05). Furthermore, the difference in PIs of CXCR1 between the group with no metastatic lymph node, the group with 1-3 metastatic lymph nodes and the group with more than three metastatic lymph nodes were statistically significant (*P* < 0.05). In addition, PI of CXCR1 in the group with negative hormone receptor was significantly higher than that with positive hormone (*P* < 0.05). Moreover, PI in Her2-positive group was obviously higher than that in her2-negative group, *P* < 0.05, (Table 1)

**Table 1:** The expression of CXCR1 was different with various clinical parameters of patients ($\bar{\text{x}}$ ± s)

Variables	Cases	Integral expression of CXCR1 in breast carcinoma tissues	*P*
Age (years)			*P* = 0.037
< 55	40	5.6 ± 0.50	
≥ 55	64	6.0 ± 1.13	
Metastatic lymph -nodes (number)			*P* = 0.000
0	50	5.5 ± 1.01	
1–3	23	6.0 ± 0.21	
> 3	31	6.7 ± 0.48	
Primary tumor size			*P* = 0.075
≥ 3 cm	61	6.0 ± 0.76	
< 3 cm	43	5.7 ± 1.11	
T stage			
T1	19	5.8 ± 0.84	P_I, II_ = 0.968
T2	67	5.7 ± 0.98	P_I, III_ = 0.001
T3	18	6.9 ± 0.45	P_II, III_ = 0.000
Differentiation of -cancer cells			
high level	19	5.7 ± 0.75	P_high, mode_ = 0.052
moderate level	37	5.8 ± 1.11	P_high, low_ = 0.003
low level	48	6.3 ± 0.96	P_mode,low_ = 0.001
ER or PR expression			
positive	62	5.5 ± 0.97	*P* = 0.01
negative	42	6.2 ± 0.84	
Her-2			
positive	25	6.3 ± 1.01	*P* = 0.043
negative	79	5.2 ± 0.87	

Note: All measurement data was expressed in the formation as $\bar{\text{x}}$ ± SD. Independent- samples *t*- test was used to analyze the expression of CXCR1 in different clinical features. *P* < 0.05 was considered statistically significant.

### The correlation between the expression change in CXCR1 before and after neo-adjuvant chemotherapy and the efficacy of neo-adjuvant chemotherapy

**Figure 3:**
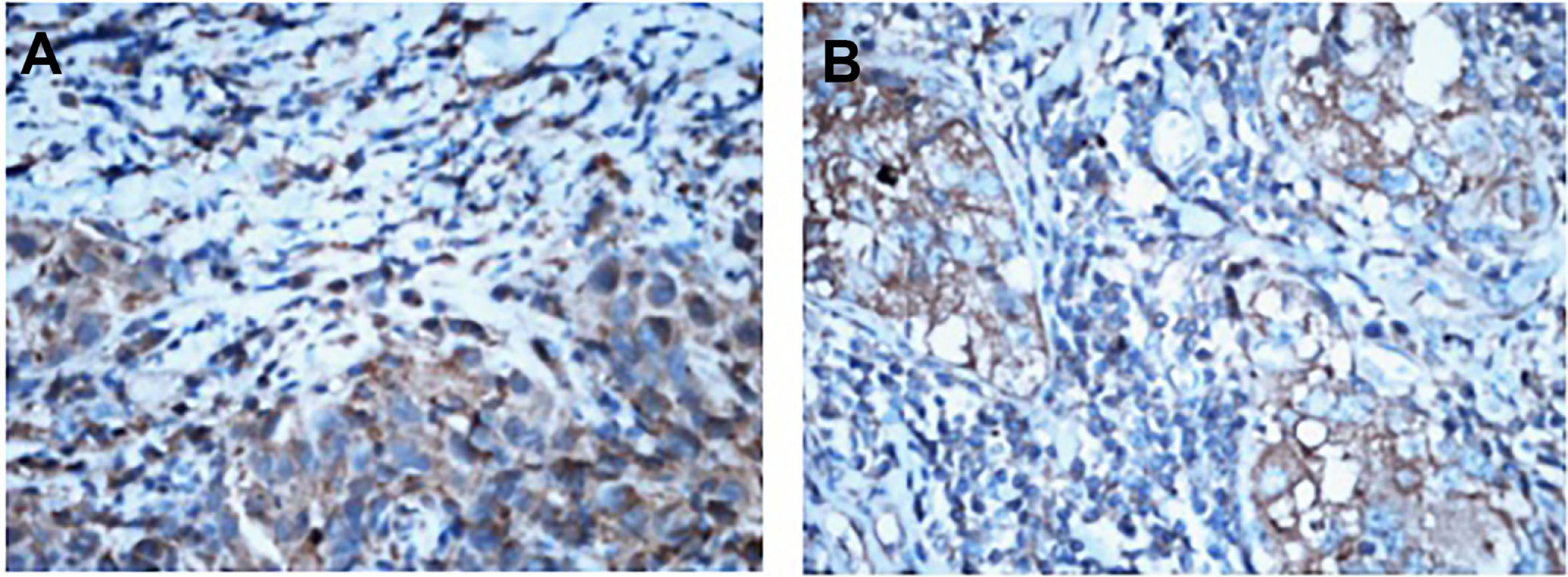
Different expression of CXCR1 in breast cancer tissues before and after neo-adjuvant chemotherapy (IHC,×400) Note: (**A**) CXCR1 expression in breast cancer tissues before neo-adjuvant chemotherapy. (**B**) CXCR1 expression in breast cancer tissues after neo-adjuvant chemotherapy.

The expression of chemokine receptor CXCR1 in biopsies of breast carcinoma was not correlated with the reaction to neo-adjuvant chemotherapy (*P* > 0.05), while the expression in surgical specimens was related to the reaction to chemotherapy (*P* < 0.05). Compared with biopsies, the PI of CXCR1 in surgical specimens highly decreased (Figure 3). The more the PI decreased, the severer the pathological reaction to chemotherapy, and the better the efficacy of neo-adjuvant chemotherapy. In the group with mild reaction to chemotherapy, the difference in PI of CXCR1 between before and after chemotherapy was not statistically significant (*P* > 0.05). However it was significant in the group with moderate reaction to chemotherapy (*P* < 0.05), also statistically significant in the group with severe reaction (*P* < 0.01, Table 2).

**Table 2:** The expression of CXCR1 declined after neo-adjuvant chemotherapy. ($\bar{\text{x}}$ ± ss)

Pathological response to chemotherapy	Efficacy of neo-adjuvant chemotherapy	Cases	Integral expression of CXCR1 in biopsy specimens	Integral expression of CXCR1 in surgical specimens
mild	general	29	5.5 ± 0.88	5.4 ± 0.72
moderate	better	27	6.0 ± 0.83	5.7 ± 0.96^Δ*^
severe	best	48	6.0 ± 1.01	3.5 ± 1.52^*^

Note: ^Δ^*P* < 0.05, compared with the group of severe response to chemotherapy; *P < 0.05,^*^P < 0.01, compared with biopsy specimens.

All measurement data were assessed using the two-sample matching test and single-factor analysis of variance, and was expressed in the formation as $\bar{\text{x}}$ ± SD. In the single-factor analysis of variance, the least significant difference (LSD) was used to compare every two groups. *P* < 0.05 was considered statistically significant.

### Follow-up result

All 104 patients were followed up, and the median follow-up period was 60 months (range: 15–99 months). Postoperative immunohistochemical results revealed that 54 patients were Luminal A breast cancer, 8 patients were Luminal B breast cancer, 17 were HER2+ breast cancer and 25 patients were triple-negative breast cancer (TNBC). A total of 11 cases suffered from local recurrence during the follow-up period, with a five-year total local recurrence rate of 10.6%. Among these cases, 2 cases were Luminal A breast cancer, 1 case was Luminal B breast cancer, 3 cases were HER2+ breast cancer and 5 cases were TNBC. These accounted for 3.7%, 12.5%, 17.6% and 20.0% of each type. Furthermore, 11 patients suffered from distant metastasis during the follow-up period, with a metastasis rate of 10.6%. Among these patients,2 cases were Luminal A breast cancer, 1 case was Luminal B breast cancer, 3 cases were HER2+ breast cancer and 6 cases were TNBC, which accounted for 1.9%, 12.5%, 17.6% and 24.0% of each type. Moreover, 1 patient of Luminal A type, 1 patient of Luminal B type, 3 patients of HER2+ type and 5 patients of TNBC died during the study period, which accounted for 1.9%, 12.5%, 17.6% and 20.0% of each type. The overall survival rate was 90.4% (Table 3).

**Table 3:** The prognosis of different molecular subtypes of breast cancer was different

Prognosis	Total cases of each prognosis	The expression of CXCR1in each prognosis	Cases of Luminal A breast cancer	Cases of Luminal B breast cancer	Cases of HER2+ breast cancer	Cases of TNBC
local recurrence	11	5.9 ± 0.41	2	1	3	5
distant metastasis	11	6.2 ± 0.37	1	1	3	6
death	10	6.6 ± 1.05	1	1	3	5
survival rate	__	__	98.1%	87.5%	82.3%	80%

Note: The case of breast cancer patients with different molecular subtypes and the expression of CXCR1 in each prognosis were expressed, and the survival rate was calculated.

Among the breast cancer patients with different molecular subtypes, the survival rate of Luminal A breast cancer was the highest, followed by Luminal B breast cancer, and the prognosis of TNBC was significantly worse than that of the other types (Table 4).

**Table 4:** Prognostic data of different molecular subtypes [n (%)]

Prognosis	Luminal A	Luminal B	HER2+	TNBC
local recurrence	2	1	3	5
distant metastasis	1	1	3	6
death	1	1	3	5
Total number of cases	54	8	17	25
survival rate	98.1	87.5	82.3	80

Note: The prognosis of patients with different molecular subtypes was different, and their survival rate was calculated.

## DISCUSSION

The development of breast cancer is an extremely complex process, which is possibly associated with a variety of oncogenes, tumor suppressor genes, and transcription factors. However, its exact mechanism remains not fully understood. In recent years, the role of chemokines, which is a kind of pro-inflammatory cytokine [5, 6], has received extensive attention in the development of tumors. Numerous studies [7, 8] have confirmed that kinds of tumors can autocrine CXC chemokines and their receptors. These secreted products may have anti-tumor effects by activating immune cells or inhibiting angiogenesis [9]. Furthermore, they could also have a direct effect in enhancing motor abilities and chemotaxis in tumor cells, as well as having indirect effect in promoting angiogenesis and the digestion of the extracellular matrix to promote tumor growth and metastasis by stimulating tumor growth [10]. It has been reported [11–14] that the use of CXCR1/CXCR2-specific antibodies *in vitro* can inhibit melanoma tumor growth.

To date, there are at least 20 kinds of identified chemokine receptors [15]. Among these receptors, CXCR1 has received more research. It had been reported in literature that CXCR1 was widely expressed in prostate cancer, bladder cancer, stomach cancer, colon cancer, endometrial cancer and melanoma cancer cells [16]. At the same time, articles also have been reported that CXCR1 plays an important role in the development and treatment of breast cancer [2, 4]. This study detected the expression of CXCR1 in normal breast tissue, breast fibroadenoma and breast carcinoma by using immunohistochemical method. Immunohistochemical results revealed that chemokine receptor CXCR1 expressed in different degrees. Only few cells in normal breast tissues expressed CXCR1 with junior staining, and the percentage of positive cells increased in breast fibroadenoma tissues. Meanwhile, almost all breast cancer cells expressed chemokine receptor CXCR1 with deep staining. We analyzed the PIs of expression of CXCR1 in each biopsy and surgical specimen and found that its expression in breast cancer was higher than that in normal breast tissue and breast fibroadenoma. Chemokine receptor CXCR1 is lowly expressed in normal breast tissue and breast fibroadenoma, but highly expressed in breast cancer, hinting that it might be used as an indicator to predict benign or malignant breast disease.

A total of 104 breast cancer patients were divided into different groups based on age, primary tumor size, pathological stage, cell differentiation, metastatic lymph node number, status of hormone receptor, the expression of Her2 and other clinical parameters, and were compared with each other in each group. It was found that the expression of CXCR1 in breast cancer tissues revealed no correlation with age and the size of the primary tumor, but revealed significant differences with the stage of pathology, degree of cell differentiation, metastasis of the lymph node, status of the hormone receptor and the expression of Her2.

The expression of CXCR1 in breast cancer tissues was higher in patients with negative hormone receptors, positive Her2, worse cell differentiation, more lymph node metastasis and later pathology stage. Meanwhile these patients had worse prognosis. These results suggest that chemokine receptor CXCR1 could auxiliarily predict the degree of malignancy of breast cancer, as well as its invasive ability and prognosis.

We followed up the 104 patients with breast cancer, and statistical results revealed that among these breast cancer patients with different molecular subtypes, the survival rate for patients with Luminal A best cancer was the highest, followed by the Luminal B breast cancer, and the prognosis for TNBC was significantly worse than that of the other types. This conclusion is consistent with previous studies [17, 18].

Chemokines and their receptors have a bi-directional natural role in the development and metastasis of tumors in both promotion and inhibition. These might have an anti-tumor effect by activating immune cells or inhibiting angiogenesis. However, in the meanwhile, these could have a direct effect in increasing motor abilities and chemotaxis in tumor cells, as well as having indirect effect in promoting angiogenesis and the digestion of the extracellular matrix, in order to promote tumor growth and metastasis by stimulating tumor growth [19]. This natural role should be considered when chemokines are applied in tumor treatment. The use of a receptor antagonist had the ability to suppress the growth and metastasis of tumors. For example, Wileu *et al*. [20] found that CCL21 acting on CCR7(+)B16 melanoma can suppress the metastasis of tumors. All of these provided new ideas.

This study found that after neo-adjuvant chemotherapy, the expression of CXCR1 in breast carcinoma decreased. The higher it decreased, the more severe the pathological reaction, and the better the efficacy of neo-adjuvant chemotherapy. In other words, the expression of chemokine receptor CXCR1 had been greatly decreasing in the process of neo-adjuvant chemotherapy for breast cancer, and there was a positive correlation between the expression of CXCR1 and neo-adjuvant chemotherapy response. If CXCR1 blockers were applied to inhibit its expression, the therapeutic response might be further improved. Scientists in the Comprehensive Cancer Center of Michigan University revealed that there was an association between breast cancer stem cells and inflammation in new studies, and chemokine receptor CXCR1 was also identified on the surface of breast cancer stem cells, which had the ability to stimulate cancer stem cell growth under the stimulation of tissue breakdown or inflammation. CXCR1 was a receptor of IL-8, which often generated in the process of chronic inflammation and tissue injury [21]. When patients who suffered from breast cancer underwent chemotherapy, the dead cells would produce IL-8, which would further promote the replication of cancer stem cells that contributing to the recurrence and metastasis of breast cancer [22]. A mouse model for human breast cancer in this study revealed that during the therapy of breast cancer, adding drugs to block CXCR1 would help kill breast cancer stem cells to improve treatment effect. Recently Laura Brandolini et al. [2] have also found that Reparixin, a powerful CXCR1 inhibitor, was effective in reducing the development of tumor and its recurrence. There is a broad prospect for the study of CXCR1, which might become an effective target for anti-tumor therapy. It is necessary to study and explore the role of CXCR1 in the development of breast cancer and the feasibility of this targeted therapy. Once it is widely used in the clinic, it would be another epoch-making breakthrough in the history of malignant tumor therapy.

## MATERIALS AND METHODS

### Specimen source

We prospectively evaluated 104 female patients with the mean age of 55.30 ± 9.14 years, who suffered invasive breast cancer. These patients were treated in NanJing Drum Tower Hospital, the Affiliated Hospital of Nanjing University Medical School, from January 2008 to December 2012. All patients were diagnosed by core needle biopsy and underwent neo-adjuvant chemotherapy according to NCCN guidelines. Based on the assessment of clinical status, 3–4 cycles of chemotherapy were carried out before performing the modified radical mastectomy of breast cancer or breast conserving surgery. We collected pre-operative biopsy specimens and post-operation paraffin specimens (including the primary tumor) of the above-mentioned 104 patients and 20 specimens with normal breast tissue (distance to the primary tumor > 10 cm). Furthermore, 20 cases with breast fibroadenoma were also collected during the same period.

### Main reagents

Concentrating rabbit anti-human CXCR1 polyclonal antibody (NBP1-88143) and the matching secondary antibody were purchased from NOVUS USA. DAB chromogenic reagent, highly sensitive washing buffer-PBS buffer system (powder), and antigen repair liquid (AR0026) were both bought from WuHan Boster Biotechnology.

### Immunohistochemical SP staining

The paraffin sections of above specimens were dewaxed, hydrated and antigen-repaired. CXCR1 antibody and antibody diluent were diluted at 1:40 according to NBP1-88143 instructions. Each section was placed in a refrigerator at 4°C overnight after adding 100 μl of the primary antibody. The matching secondary antibody was added the next day and was set in an incubation temperature box in the following day. Next, we began DAB dyeing under a microscope following the dehydration and sealing piece. And phosphate-buffered saline (PBS) instead of the primary antibody was chose as the negative control.

### Reading of sections under the microscope (×400 times)

Five fields of vision were taken from section. The score was given on the basis of the percentage of positive cells and staining intensity in each vision. The score of the percentage of positive cells was assigned based on the proportion of positive cells in view of total cell population as follows: < 10% for 0 points, 10% ∼ 25% for 1 point, 26% ∼ 50% for 2 points, 50% ∼ 75% for 3 points and > 75% for 4 points. The staining intensity score was assigned by dyed color as follows: positive cells without coloring noted for 0 points, dyed pale yellow noted for 1 point, dyed tan noted for 2 points, dyed brown noted for 3 points. These two scores were then summed as the positive integral (PI).

### Pathological classification of neo-adjuvant chemotherapy for malignant tumors

The surgical specimens were stained with hematoxylin and eosin (H&E) and the organizational structure was observed under an Olympus microscope. The surgical specimens were grated according to Miller and Payne classification as follows: Grades 1 and 2 noted for mild response to chemotherapy, Grade 3 noted for moderate response, and Grades 4 & 5 noted for severe response. The heavier the pathological reaction to chemotherapy, chemotherapy curative effect was better.

### Follow-up method

According to NCCN guidelines, all 104 breast cancer patients received postoperative therapies such as chemotherapy, radiation and hormone therapy. They received follow-up from the day of discharge at the end of the operation, every three months in three years, and every six months three years later. Breast cancer recurrence or distant metastasis could be found by clinical and histological examinations. All patients received telephone follow-up every six months until June in 2016 or the patient's death.

### Statistical method

All statistical analysis was performed using statistical software SPSS 19.0. All measurement data were assessed using the two-sample matching test and single-factor analysis of variance, and was expressed in the formation as x ± SD. In the single-factor analysis of variance, the least significant difference (LSD) was used to compare every two groups. *P* < 0.05 was considered statistically significant.

## Abbreviations

PBS: phosphate-buffered saline
PI: positive integral
H&E: hematoxylin and eosin
LSD: least significant difference
TNBC: triple-negative breast cancer
